# Efficient and surface site-selective ion desorption by positron annihilation

**DOI:** 10.1038/s41598-018-25506-5

**Published:** 2018-05-08

**Authors:** Takayuki Tachibana, Takashi Yamashita, Masaru Nagira, Hisakuni Yabuki, Yasuyuki Nagashima

**Affiliations:** Department of Physics, Tokyo University of Science, 1-3 Kagurazaka, Shinjuku Tokyo, 162-8601 Japan

## Abstract

We compared positron- and electron-stimulated desorption (e^+^SD and ESD) of positive ions from a TiO_2_(110) surface. Although desorption of O^+^ ions was observed in both experiments, the desorption efficiency caused by positron bombardment was larger by one order of magnitude than that caused by electron bombardment at an incident energy of 500 eV. e^+^SD of O^+^ ions remained highly efficient with incident positron energies between 10 eV and 600 eV. The results indicate that e^+^SD of O^+^ ions is predominantly caused by pair annihilation of surface-trapped positrons with inner-shell electrons. We also tested e^+^SD from water chemisorbed on the TiO_2_ surface and found that the desorption of specific ions was enhanced by positron annihilation, above the ion yield with electron bombardment. This finding corroborates our conclusion that annihilation-site selectivity of positrons results in site-selective ion desorption from a bombarded surface.

## Introduction

Positron annihilation techniques are recognised to be non-destructive and are extensively used to investigate electronic states in metals^1^, vacancy-type defects in solids^2^, crystal structures of solid surfaces^3^ and chemical states of surface layers^4^. The positrons injected into materials may break the atomic bonds via pair-annihilation^5^. Additionally, emission of Auger electrons after positron annihilation with core electrons^4^ may create a two-hole final state that causes ion desorption from the surface by Coulomb repulsion. Although there is a possibility that a solid surface will be modified by these phenomena, considerably less information about these processes is available.

Desorption of atoms, molecules and ions from solid surfaces following electronic transitions that were induced by incident electrons or photons, referred to as desorption induced by electronic transitions (DIET), has been actively studied^6,7^. This phenomenon provides a powerful tool to study fundamental surface processes and achieve various technological applications, for example, in the areas of modification of semiconductor devices, radiation biology and development of surface spectroscopic techniques^8–10^.

Recently, we observed positron-stimulated desorption (e^+^SD) from a TiO_2_ surface^11–13^, one of the canonical targets for investigating DIET processes. Desorbed O^+^ ions were clearly observed at an incident positron energy below the desorption threshold by electron impact and photon absorption. This indicates that O^+^ ion desorption is not caused by impact excitation but instead is caused by pair annihilation of incident positrons with atomic inner-shell electrons at the surface. This finding introduces the possibility of experimentally studying positron-induced surface dynamics and developing potential applications. However, the DIET processes and the specific mechanisms mediating ion desorption caused by positrons remain unclear.

In the present work, we compare e^+^SD and electron-stimulated desorption (ESD) of ions from a TiO_2_(110) surface. The results show that positron annihilation enhances specific ion desorption above the yield observed with electron bombardment. We conclude that trapping of positrons at the surface and the tendency for annihilation to occur at specific surface sites strongly affect the e^+^SD phenomenon.

## Results and Discussion

### Comparison of ESD and e^+^SD from TiO_2_ surface

**Figure 1:**
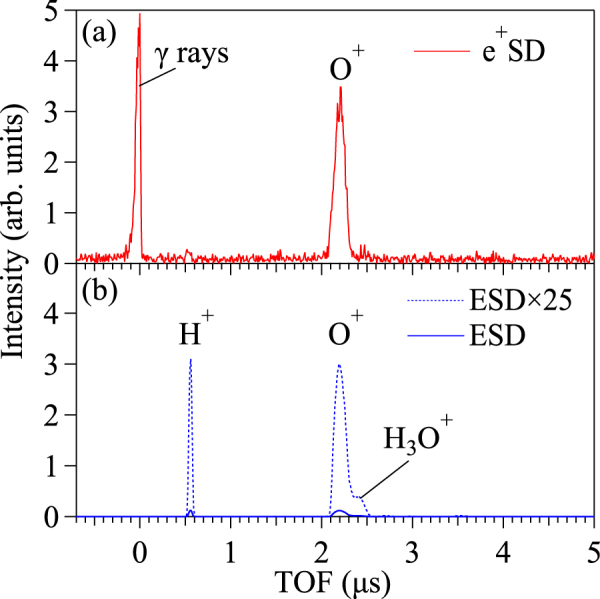
TOF spectra of ions desorbed from a TiO_2_(110) surface by (**a**) positron and (**b**) electron bombardment at an incident energy of 500 eV. Each spectrum was normalised to the number of incident particles. The blue dashed line in (**b**) indicates ESD results enlarged by a factor of 25.

The time-of-flight (TOF) spectra of cations desorbed from the TiO_2_(110) surface during irradiation with electrons and positrons are shown in Fig. 1. The incident positron energy was set to 500 eV to be equal to the electron-beam energy. Each spectrum was normalised to the number of incident particles, as estimated by the electron-beam current or the count-rate of the NaI(Tl) scintillation detector. A peak at time zero appears only in the e^+^SD-TOF spectrum and is due to the detection of γ rays emitted from the target by a micro-channel plate (MCP) ion detector.

Considerable differences in the TOF distributions of e^+^SD and ESD were found. While the desorption of O^+^ ions from the TiO_2_ lattice was observed in both measurements, the O^+^ peak intensity caused by e^+^SD was approximately 25 times larger than that caused by ESD. In contrast, H^+^ and H_3_O^+^ ions generated from a slight coverage of residual gases, typically H_2_O molecules, were produced less efficiently by e^+^SD. These dissimilar spectra indicate that the peculiar interactions of positrons with a solid surface are reflected strongly the desorption phenomena.

### Dependence of O^+^ ion yield on the incident positron energy

In DIET studies, information concerning desorption processes has typically been obtained from measurements of the threshold value of the incident energy for desorption and the desorption yield. Moreover, identifying the initial excitation states is crucial when considering subsequent electronic transition processes. In 1978, Knotek and Feibelman (KF)^14^ proposed a model for O^+^ ion desorption from TiO_2_ surface based on their observations of an ESD threshold of 34 eV, which matches the excitation energy of the Ti(3p) core level. The ionic states in ideal TiO_2_ are commonly understood to be a Ti^4+^ cation bound ionically with O^2−^ anions. Thus, the formation of O^+^ ions in the lattice requires a charge transfer of at least three electrons. The interpretation offered by Knotek and Feibelman indicated that the Ti(3p) core hole created by an electron impact is filled with an electron from the O(2p) orbitals of a neighbouring O^2−^ anion via an inter-atomic Auger process, resulting in the emission of two electrons from the valence orbitals as Auger electrons. Consequently, O^+^ ions formed from O^2−^ anions can be expelled from the surface via Coulombic repulsion from the surrounding Ti^4+^ cations. Although KF originally proposed their model to explain the desorption of O^+^ ions from TiO_2_ surfaces by the excitation of inner-shell electrons in Ti atoms, the concept of Auger-stimulated desorption can be extended to explain ion desorption from many materials. Furthermore, electron-ion coincidence spectroscopy measurements of TiO_2_ surfaces show that intra-atomic Auger decay after O(1s) excitation can also cause O^+^ ion desorption^15^.

**Figure 2:**
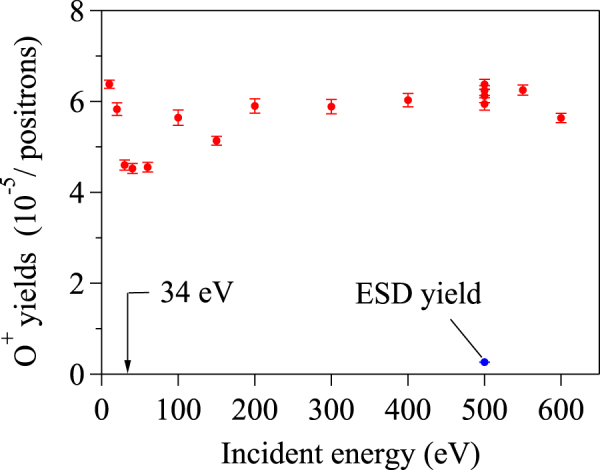
Desorption yields of O^+^ ions from a TiO_2_(110) surface as a function of incident positron energy. The arrow indicates the initial ESD threshold of 34 eV. The ESD yield of O^+^ ions by a 500-eV electron beam is also plotted and marked.

Based on these previously reported ESD threshold energies and ion-yield curves, our study of the dependence of e^+^SD on incident positron energy offers further insight. Figure 2 plots the e^+^SD yields of O^+^ ions from the TiO_2_ surface as a function of incident energy in the range between 10 eV and 600 eV. Surprisingly, the measured values remained nearly constant even with such a wide range of incident energies. This striking feature is quite different from the ESD-yield curves, which strongly depend on the incident electron energy. For example, ESD of O^+^ ions does not occur below the threshold of 34 eV^14^, whereas the yield curve rises sharply just above the threshold and then tends to increase with increasing incident energy that ranges from the threshold to 500 eV^7^. This relation depends on factors such as energy dependence of the initial excitation cross section, the final accessible states and dissipation channels of energy deposition. Additionally, the secondary electrons that originate from the bulk can also contribute to the desorption yields^7^. The scattering interaction of primary electrons with the bulk atoms causes the production of multiple secondary electrons. Their kinetic energies are lower than the incident energy of the primary electrons. However, secondary electrons still possess sufficient energies to induce inner-shell excitations when primary electrons having incident energy that is considerably greater than the threshold are injected into the target. Some of the secondary electrons may strike the outermost surface layer and cause stimulated desorption. The ESD yields of the desorbed O^+^ ions from the TiO_2_ surface are enhanced because of the secondary ESD effect, especially at an incident energy that approximately ranges from 110 to 180 eV, in which no corresponding core levels are observed to exist for Ti or O atoms. Owing to their contributions of primary and secondary electrons, the yield at an incident energy of 500 eV is observed to be larger than the yield from incident energies near the threshold by several orders of magnitude^7^. Although the ratio of the e^+^SD O^+^ ion yield to that of ESD is approximately 25:1 at the incident energy of 500 eV, it is much more pronounced at lower incident energies.

If e^+^SD is mediated by impact excitations, the desorption yield would depend on the incident positron energy, as it does with ESD. Therefore, the nearly constant e^+^SD yield of O^+^ ions cannot be explained by the impact-excitation process. This also suggests that e^+^SD of O^+^ ions is primarily mediated by the same electronic transition process, regardless of the incident energy. In our previous e^+^SD study, we concluded that the only process leading to O^+^ ion desorption below the threshold of 34 eV is positron annihilation with core electrons at the surface^11^, which can induce Auger-stimulated desorption without consuming incident kinetic energy in inner-shell excitations. Thus, our interpretation of the present results is that e^+^SD of O^+^ ions is predominantly caused by positron annihilations, even for incident energies above the threshold. Of course, high-energy positron bombardment can also cause the desorption via the impact excitations, including the secondary ESD effect. However, these processes make less of a contribution to the O^+^ ion-yield curve, as the positron-annihilation process is highly efficient.

### Annihilation-site selectivity of surface trapping positrons

We next need to explain how the positron-annihilation process is more efficient for O^+^ ion desorption and why the O^+^ yield depends less on incident energy than it does with electron bombardment. Entrapment of diffusing positrons at the TiO_2_ surface offers one possible explanation. When positrons are injected into solids, they do not annihilate with atomic electrons immediately, but instead rapidly decelerate to thermal equilibrium via inelastic collisions and then diffuse randomly in the bulk of the solid over a time period of ≲100 ps (see Fig. 11 in ref.^1^). Because the typical diffusion length for positrons in metals and semiconductors is approximately 0.1 *µ*m, positrons in the near-surface region can diffuse back to the solid surface. In many metals, positrons that reach the surface can be trapped in surface states that are induced by the combination of their own attractive image-potential and the repulsive bulk-potential until they annihilate^1,16^. The surface states of positrons have also been studied for insulators^17,18^.

If surface states occur on the TiO_2_ surface, desorption yield caused by positron annihilation would depend on the fraction of diffusing positrons that are trapped, which is related to the bulk penetration depth. However, the fractional value should be saturated with incident energies lower than 1 keV, as most positrons can diffuse back to the surface because of the sufficiently small ratio of penetration depth to diffusion length^1^. Consequently, the e^+^SD yield of O^+^ ions does not depend on incident energy in our present results. Moreover, the positrons trapped in the surface states would cause efficient Auger-stimulated O^+^ desorption than the inner-shell excitations by the positron bombardment and the secondary ESD effect, even though the probability of annihilation with core electrons is a few percent or less, which is much lower than the probability of annihilation with valence electrons^19^. We recently investigated a positron state at the TiO_2_(110) surface using both computational and experimental methods. These results strongly support our interpretation that the diffusing positron can be localised and annihilate at the topmost surface layer^20^.

On the assumption that almost all O^+^ ions observed from e^+^SD are desorbed via positron annihilation, the differences in desorbed ion species between e^+^SD and ESD (shown in Fig. 1) may be explained by the annihilation-site selectivity of positrons. The positrons diffusing in the surface layers of alloys tend to be attracted to regions that composed one kind of atoms with a relatively higher positron affinity, where they tend to annihilate^21^. In the highly ionic rutile-TiO_2_ lattice, theoretical calculations using local density approximation have shown that positrons are localised in the interstitial regions and primarily overlap with the electrons distributed around O^2−^ anions, rather than with those around Ti^4+^ cations^22^. Accordingly, e^+^SD of O^+^ ions is most likely caused by the annihilation of surface-state positrons with the core electrons of surface oxygen atoms, although core-hole creation in both Ti and O atoms at the TiO_2_ surface can lead to O^+^ ion desorption^15,23^. In contrast, ESD of H^+^ ions has been explained by the electrically stimulated fragmentation of the hydroxyl OH groups of the Ti-OH sites formed by dissociative chemisorption of H_2_O molecules at oxygen-vacancy sites^24,25^. Each oxygen vacancy on the TiO_2_ surface results in two Ti^3+^ sites^26^. Thus, the inefficient H^+^ ion yield of e^+^SD suggests that positrons at the surface are reluctant to annihilate near OH molecules bound to Ti^3+^ sites and Ti^4+^ sites.

In addition to surface trapping, the annihilation-site selectivity of positrons may also be related to the highly efficient e^+^SD yield of O^+^ ions. The probability of desorption after inner-shell excitation for each oxygen atom at the TiO_2_(011) surface is influenced by the environment around the existing site^27^, and the excited oxygen atoms adjacent to the oxygen defects have desorption probabilities one or two orders of magnitude lower than those in the defect-free regions. Positrons are known to be sensitively trapped by vacancy defects^1^. However, positrons trapped at the TiO_2_ surface may annihilate predominately at O^2−^ sites in defect-free regions because they are repelled by the Coulomb force from oxygen vacancies, namely, the Ti^3+^ sites. This site-specificity enhances the e^+^SD yield of O^+^ ions.

### ESD and e^+^SD from water chemisorbed on TiO_2_ surface

**Figure 3:**
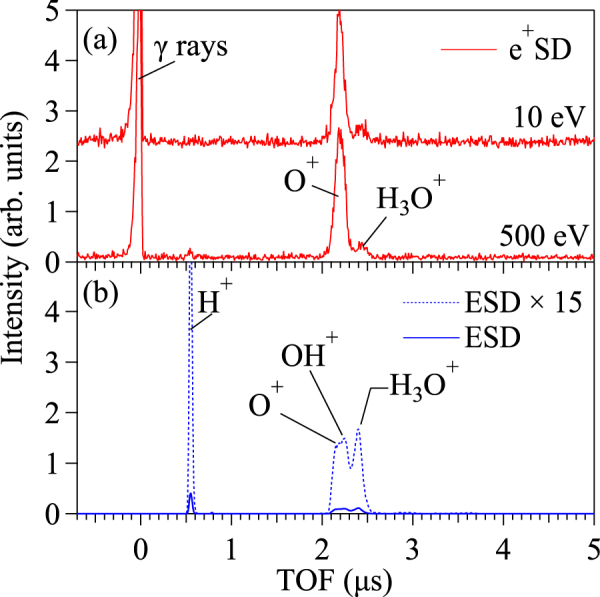
TOF spectra of the ions desorbed from water chemisorbed on a TiO_2_(110) surface caused by (**a**) positron bombardment at incident energies of 10 eV and 500 eV and TOF spectrum (**b**) 500-eV electron beam bombardment. Each spectrum was normalised to the number of incident particles. The blue dashed line in (**b**) indicates ESD results enlarged by a factor of 15.

To further probe the site-specific DIET phenomena caused by positron bombardment, e^+^SD and ESD from the water chemisorbed on the TiO_2_ surface were also observed at an incident energy of 500 eV (Fig. 3). Distilled water was introduced into the ultra-high vacuum (UHV) chamber through a variable leak valve. The TiO_2_(110) surface was exposed to water vapor at a pressure of 1 × 10^−5^ Pa for a few minutes. The ESD-TOF spectrum was noticeably affected by the water chemisorption. The signals from H^+^ and H_3_O^+^ ions increased, and the desorption of OH^+^ ions was also observed. However, O^+^ ions desorbed by e^+^SD remained dominant, even though the yield was reduced. Furthermore, a relatively small peak of H_3_O^+^ ions appeared in the e^+^SD-TOF spectra. These results clarify the site-specific ion desorption process by illustrating the annihilation-site selectivity of the positrons. ESD of OH^+^ ions has been explained by the inner-shell excited Ti-OH sites, similar to the explanation for ESD of H^+^ ions^24,25^. Thus, we expect to observe very low intensities of these ions in e^+^SD yields, considering the annihilation-site selectivity of the positrons. In contrast, the H_3_O^+^ ions observed in both ESD and e^+^SD are likely to be ejected from water molecules or surface hydroxyl water complexes. Comparable yields of H_3_O^+^ and O^+^ ions were observed from e^+^SD, even with a 10-eV incident positron beam. The ESD thresholds of H_3_O^+^ ions from an ice surface have been reported at ∼22 eV and ∼70 eV^28^, suggesting that e^+^SD of H_3_O^+^ ions from the TiO_2_ surface is also caused by positron annihilation in an analogue of the process leading to e^+^SD of O^+^ ions. The surface-trapped positrons are likely to favour annihilation at the sites of the chemisorbed water molecules, rather than at the Ti-OH sites, although the fact remains that they mostly annihilate with the electrons at O^2−^ sites.

## Conclusion

In summary, we compared e^+^SD and ESD of positive ions from a TiO_2_(110) surface. Surface trapping and annihilation-site selectivity of positrons strongly affect the desorbed ion yield and the ion species. We expect our results to stimulate future studies on atomic surface processes induced by positron annihilation, which have so far been only poorly understood. Additionally, e^+^SD can be adapted as a tool to investigate the unique behaviours of the positrons in the near-surface region. Our findings mentioned above are of fundamental importance for the fields of surface science and positron physics, as positron beams have proven useful as surface-sensitive probes over the past few decades^29^. For example, our results will provide important information related to positron-annihilation-induced Auger electron spectroscopy (PAES)^4^. PAES is generally conducted using a low energy positron beam to eliminate the large secondary electron background; however, we have depicted that the inner-shell ionization process by positron annihilation predominates the impact process, even in the high incident energy range.

The site-specific e^+^SD also has potential for applications in the field of surface modification. Auger-stimulated ion desorption by electrons or photons is likely to occur around the initially excited atomic site because tightly bound inner shell electrons are highly localised near one atomic site. Therefore, the site-specific chemical bond scission and desorption can be achieved by the selective core excitation in different atomic sites or different energy levels even in the same atom. A practical example of the site-specific desorption is to control the electronic and optic properties of oxidised materials and semiconductors that are significantly influenced by the structural defects and vacancies. However, secondary ESD effect is a serious limitation to perform site-specific desorption that is related to the excitation of deep core levels^30^. The secondary electrons can randomly collide with the surface layer and cause non-selective excitations. Therefore, site-specific desorption using a low energy beam is desirable but has proven to be challenging while conducting DIET studies. Recently, a diffraction technique in ESD that includes spherical-wave effects and multiple scattering of low energy incident electrons was developed, which demonstrated a high ability to extract site-specific information, especially at near the desorption threshold^31^. The site-selective e^+^SD caused due to the surface trapping and the annihilation-site selectivity of positrons may be proposed a different technique and may be useful to control the surface reactivities when conventional ESD-based methods are impractical.

## Methods

### Experimental setup

**Figure 4:**
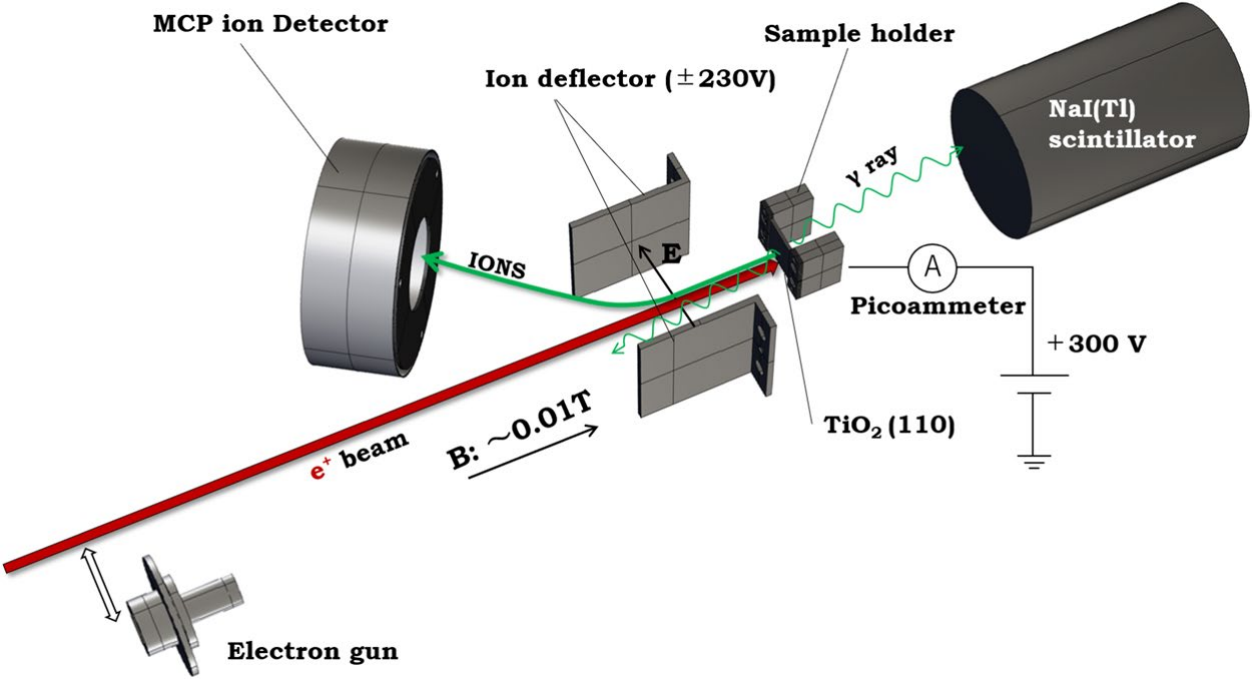
Schematic diagram of the TOF measurement system for desorbed ions. A positron beam was transported by an axial magnetic field (0.01 T) and was incident normal to the sample surface. The TiO_2_ sample was biased at a voltage of 300 V. Annihilation γ rays emitted from the target were detected by a NaI(Tl) scintillator coupled with a photomultiplier tube. The positive ions desorbed from the target were accelerated by the target bias of 300 V and were directed to an ion deflector with a potential difference of 460 V. The ionic trajectories in the deflector were deflected and directed to a MCP ion detector. The e^+^SD-TOF spectrum was obtained through analysis of the time interval between the signals from the NaI(Tl) scintillator and the MCP. An electron gun was mounted on a linear manipulator to move it into and out of the beam path, allowing measurement of the ESD-TOF spectrum.

All experiments were performed in an UHV chamber with a base pressure of less than 3 × 10^−8^ Pa. The chamber was equipped with a molybdenum sample holder, a modified TOF analyser^11^ and an electron gun (Fig. 4). Oxygen gas and distilled water were introduced into the chamber through two independent gas lines fitted with a variable leak valve. The distilled water was purified by multiple freeze-pump-thaw cycles. The surface of the rutile TiO_2_(110) (Shinkosha, 15 mm × 15 mm × 0.5 mm) was mounted on a silicon wafer for resistive heating. The crystal was cleaned with a heating cycle at 900 K. After this initial step, the surface was re-oxidised at 900 K in the presence of oxygen at a pressure of 1 × 10^−4^ Pa.

### e^+^SD-TOF system

A variable-energy positron beam produced from a ^22^Na source (275 MBq) was guided from the system to the target using an axial magnetic field (0.01 T) and passed through an ion deflector formed by a pair of parallel plates with a potential difference of 460 V. The beam trajectory drifted slightly in that region and was then directed to the TiO_2_ sample. A NaI(Tl) scintillation detector was placed behind the target to monitor the 511-keV annihilation γ rays emitted from the target. The intensity of the positron beam at the target position was approximately 2 × 10^4^ s^−1^. Under the magnetic field used for beam transport, the ions desorbed from the target were detected using a TOF analyser. Because a bias voltage of 300 V was applied to the target, the ions desorbed from the surface were accelerated toward the ion deflector. The radius of cyclotron motion for the ions in the magnetic field is correspondingly larger than that for positrons because of their heavy mass. Thus, the ion trajectories in the electric field between the deflector plates are strongly deflected and directed to the MCP ion detector, which was placed at an angle of 35° with respect to the beam axis. The outputs of the MCP and NaI(Tl) detectors were connected to a high-speed digitiser (National Instruments, NI PCI-5152). The e^+^SD-TOF spectrum was obtained through analysis the time interval between the input signals from both the detectors.

### ESD-TOF system

For comparison with ESD, the electron gun provided a 200-eV pulsed electron beam with a duration of 30 ns at a repetition rate of 1 kHz. The incident electron energy to the 300-V biased sample was 500 eV. The electron-beam current to the sample was measured using a floating picoammeter (Keithley, 6487). The intensity of the electron beam was approximately 1 × 10^8^ s^−1^. Although more intense electron beams could be easily obtained using the electron gun, it may result in charge-up of the target surface. We maintained a low beam intensity during the ESD measurements, which were of limited value to accurately measure the beam current that was observed in our system. Nevertheless, the electron beam intensity is considerably higher than the positron beam intensity, which is approximately 2 × 10^4^ s^−1^, that is measured using a scintillation γ ray detector. We therefore checked the dependence of the electron beam intensities on the ESD ion yields and confirmed that the yields are observed to linearly depend on the increasing beam intensity in the range of a maximum of 100 times the value that was used in the ESD measurement. This indicates that the contribution of the charge-up effect is negligible while performing our measurements. The gun was mounted on a linear manipulator to move it into and out of the beam path, allowing us to observe both the ESD ions and e^+^SD ions from the same sample without breaking the UHV. For the ESD measurements, waveform signals from the MCP detector were stored in the digitiser when triggered by an external signal, which was synchronised with the pulse repetition rate.

### Data availability

All data generated or analysed during this study are included in this published article.
